# A mechanical insight into the triggering mechanism of frequently occurred landslides along the contact between loess and red clay

**DOI:** 10.1038/s41598-021-96384-7

**Published:** 2021-09-02

**Authors:** Baoqin Lian, Xingang Wang, Kai Liu, Sheng Hu, Xiao Feng

**Affiliations:** 1 State Key Laboratory of Continental Dynamics, Department of Geology, Northwest University, Xi’an, 710069 China; 2 College of Urban and Environmental Science, Northwest University, Xi’an, 710127 China; 3 State Key Laboratory of Petroleum Resources and Prospecting, China University of Petroleum (Beijing), Beijing, 102249 China

**Keywords:** Natural hazards, Materials science

## Abstract

The triggering mechanism and movement evolution of loess-red clay landslides, which occurred frequently along the contact between the loess and red clay on the Loess Plateau, are closely related to the mechanical properties of the contact surface. This work presents an experimental investigation on loess, clay and loess-red clay interlaminar (LRCI) samples obtained from a typical loess-red clay landslide in northern part of Shaanxi province of China, using a series of ring shear tests, microscopic observation and scanning electron microscopy tests, in an attempt to explore the mechanical behavior of loess, clay and LRCI samples with variation in moisture content, normal stress and shear rate. The results revealed that for all specimens, both the peak shear strength $\tau_{p}$ and the residual shear strength $\tau_{r}$ decreased with increasing moisture content, among which, moisture content has the greatest influence on the $\tau_{p}$ and $\tau_{r}$ of red clay, followed by the LRCI specimen, and the loess specimen is least affected by moisture content. Meanwhile, exponential functions describing the correlations between shear strength and moisture content of LRCI, red clay and loess specimens were proposed. Furthermore, the macroscopic morphological characteristics and the microstructure of shear surface obtained from the LRCI specimens showed that a localized water accumulation was built up within the shear surface as the water content increases to some extent, and a high degree of liquefaction developed within shear surface when the moisture content reached to the saturate degree. The microstructural observation on LRCI specimen suggested that the shear surface became smoother and the larger percentage of small-sized pores was observed with moisture content. Accordingly, the built-up excess pore water pressure during shearing is difficult to be dissipated due to a close structure of small-sized pores. Due to the low permeability, high pore-water pressure built up within the shear zone and the increase in the fine particle content, the LRCI soils with a high saturation degree shows the potential for the localized liquefaction within shear zone, which further provides a scientific explanation for the triggering mechanism of loess-red clay landslides with high-speed and long- run out.

## Introduction

Loess landslides have been recognized as one of the most common geologic hazards on the Loess Plateau of China^1,2^. According to the composition of the slide mass and the situation of the failure plane, four types of landslide have been identified: ‘slides within loess’, ‘bedrock contact landslides’, ‘palaeosol contact landslides’ and ‘mixed landslides’^3–5^. Among four types of loess landslides, landslides occur along the contact between loess soils and red clay make up the most percentage of landslides throughout the Chinese Loess Plateau^3^. Some catastrophic loess landslides occurring along the contact between loess soils and red clay had been reported in China, causing severe casualty almost every year^6,7^.

Catastrophic loess landslides can occur in various soil types, such as loess soils, residual soils and sensitive clays. It is generally understood that these loess landslides are related to the decrease in the shear strength of slip zone soils. It has been found that the characteristics of slip zone soils plays a key role in the evolution of a loess landslide^8–10^. The contact interface between loess soils and red clay easily develops into a weak surface, which would eventually evolve into a sliding surface^11,12^, affecting the occurrence of landslides^3,13^. For example, it was reported that the average area and length of red clay contact landslides are 5.52 and 2.45 times larger than those of landslides occurred within loess^14^, and numerous researchers have pointed out that the mechanical characteristics of a contact surface between loess soils and red clay^2,6,15^ is a basic factor controlling the occurrence frequency and extent of a Loess-Red clay landslide on the Loess Plateau^10,16,17^. Therefore, understanding the mechanical properties of slip zone soils is of great significance to get deeper insight into the triggering mechanism of a loess-red clay landslide^12,18^.

To date, there has been considerable research into understanding the mechanical properties of slip zone soils with main efforts on examining the shear behavior of slip zone soils collected from loess landslides^19–21^. There have also many attempts to investigate the shear behavior of slip zone soils^11,22^, with main focus on the landslides occurred along the paleosol contact surface. These researches enabled us to understand the basic mechanical behavior of slip zone soils, and some factors influencing the evolution and movement of landslides. However, compared to those slip zone soils obtained from loess landslides and paleosoil contact landslides, the studies on the mechanical characteristics of slip zone soils of loess-red clay landslides are rare, but have been reported by some researchers^13^. In previous studies, Wen, Wang^23^ explored the deformation process of a loess-red clay landslide by using the field monitoring and laboratory physical modeling and found that rainfall has a significant impact on the deformation characteristics of slopes through its interaction contact between the loess soil and the loess-Neogene red mudstone, while the variation in the residual strength of slip zone soils during the shearing process was not mentioned. Using the pipette method and fine X-ray diffraction method, Shi et al.^7^ pointed out that rainfall gradually destroyed the structure of the Neogene clay and transformed it into slip zone soils, which causes the shear strength of the Neogene clay to decrease and the ground water table level to rise as well. However, the mechanical properties of slip zone soils was not investigated. Furthermore, Zhang et al.^10^ investigated the shear surface behavior of red clay samples obtained from loess-red clay landslides through a series of dynamic triaxial tests and reported that the shear strength of clay degenerated more in the cyclic triaxial tests compared to that in the static triaxial tests, while microstructure characteristics of soils was not mentioned, and the advanced apparatus which could better determine the strength parameters of slip zone soils was not been used^24,25^. The review of the literature enabled us to know that the mechanical characteristics of slip zone soils as well as the factors affecting the occurrences of loess-red clay landslides still requires experimental evidence.

To better examine triggering mechanism of landslides occurred along the contact between loess and red clay, Loess-red clay interlaminar (LRCI) samples were used to conduct a series of ring shear tests in this study. In addition, ring shear tests results using loess samples and red clay samples were compared with that using LRCI samples. Furthermore, the effect of moisture content (Mc), normal stress ($\sigma_{n}$) and shear rate ($v$) on the shear strength was concluded and the relationships between shear strength and moisture content were obtained for LRCI, red clay and loess specimen. Finally, the influence of moisture content on the macroscopic morphology, microstructure characteristics and quantitative micro-pore parameters of the shear surface of LRCI specimen were discussed as well. The result is apt to provide experimental and theoretical basis for interpreting the triggering mechanism of such kind landslides, predicting the landslide evolution, and handling loess-red clay landslide problems.

## Materials and Methods

### Testing sample

**Figure 1:**
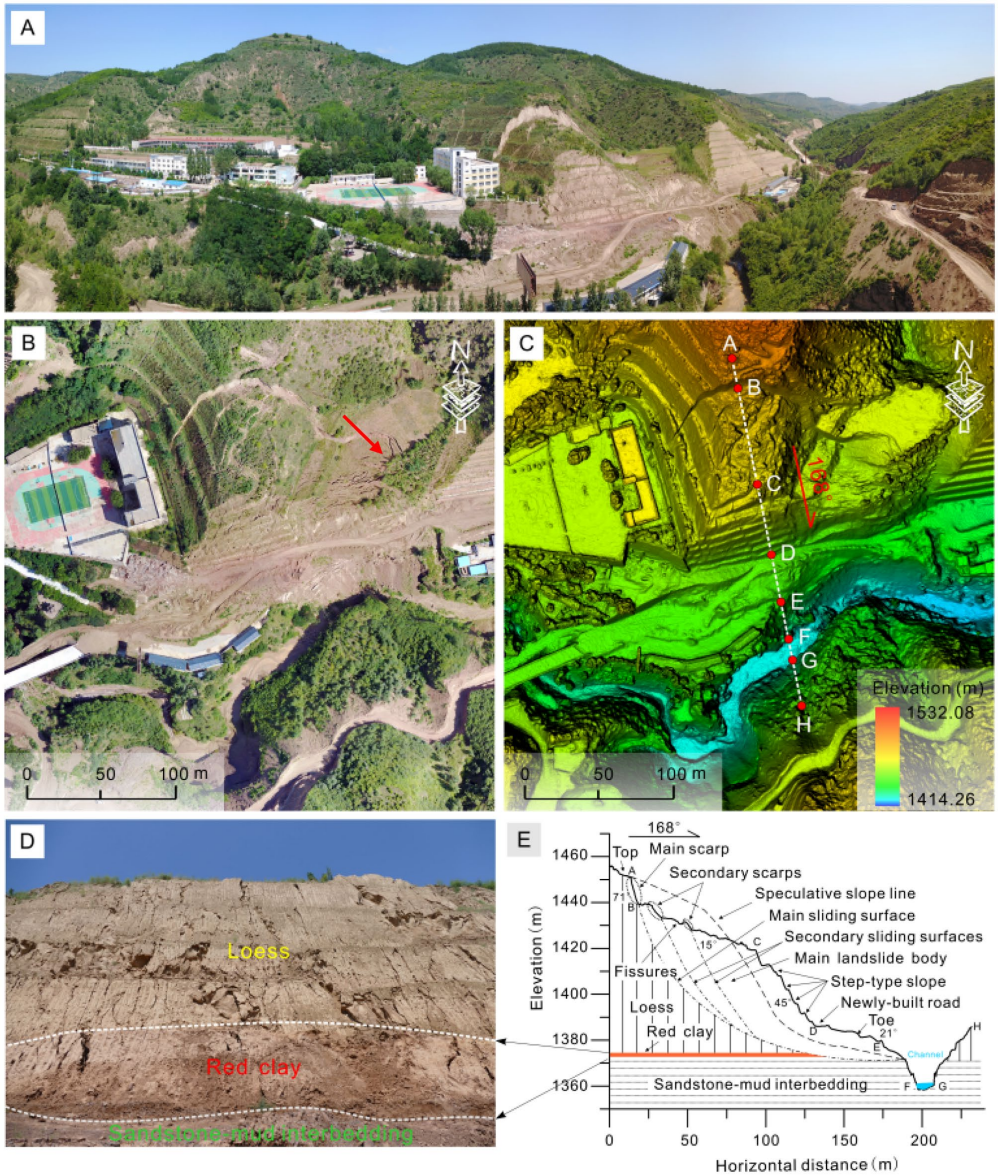
Loess-red clay landslide in Zhifang village elementary school. (**a**) Overview of landslide; (**b**) landslide crack; (**c**) DEM image of landslide; (**d**) landslide stratum; (**e**) sectional view of the main sliding direction of the landslide.

**Figure 2:**
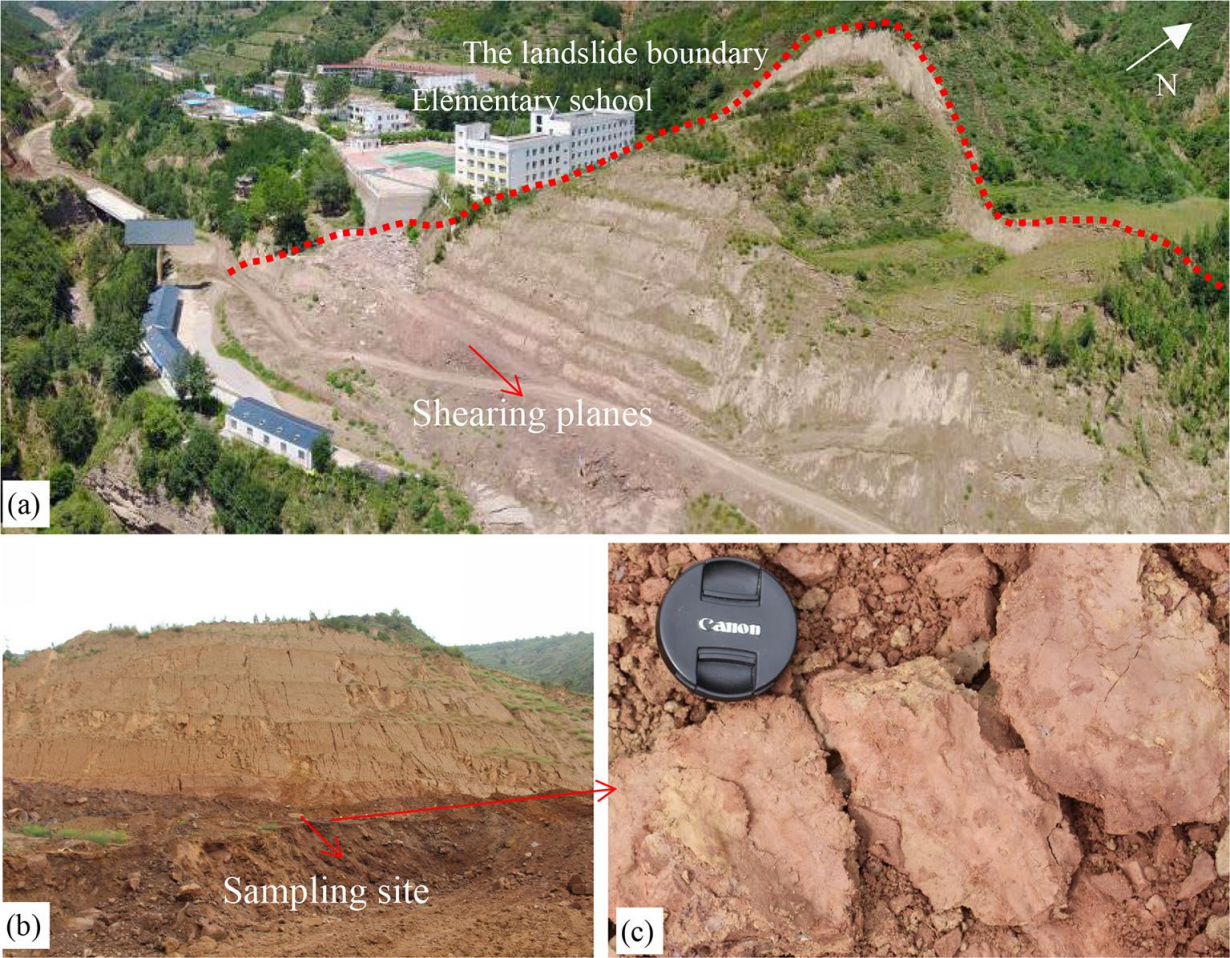
Sliding scratch surfaces and sampling site. (**a**) The landslide boundary; (**b**) sampling site; (**c**) sliding scratch surfaces.

Disturbed soil samples used in this study were taken from a landslide (36.84°N, 108.61°E) that occurred at an elementary school in Zhidan County, Shaanxi, China, which is located in the hinterland of the Loess Plateau (Fig. 1a). The landslide is recognized as a catastrophic landslides due to its high speed and long run-out distance^26^. The upper part of the sliding mass is covered with a thick layer of loess that is characterized by lots of pores and well-developed cracks (see the red arrow in Fig. 1b). The main direction of the landslide is 168° north to east (Fig. 1c). In the lower part of the sliding mass, a dense structure of Pliocene red clay was found, with a thickness of about 3 m to 6 m (Fig. 1d). The sliding mass with the average slope 41° has a relative height difference of 103 m (Fig. 1e), the length of 174 m and the width of 172 m, covering area approximately 24,080 m^2^^26^. The red clay has been identified as slip zone soil as the sliding scratches are obviously visible on the surface of the red clay at the slip zone (Fig. 2). Table 1 lists the physical parameters of loess and red clay which were collected for testing. As for red clay samples, clay content accounts for about 56.4% and main mineral components such as montmorillonite and illite are dominated. The red clay appears dark red as it contains relatively more proportions of free iron oxide, indicating that the red clay is formed in semi-arid and humid climate environment. The collected loess and red clay samples were completely air-dried, crushed and sieved through a 2 mm mesh screen, and then kept in an oven at 105 °C for more than 24 h to fully dry for sample preparation.

**Table 1:** Physical parameters of Lishi loess used in the test.

*Material*	*ρ*	*ρ*_*d*_	*W*_*n*_	*W*_*s*_	*W*_*L*_	*W*_p_
Loess	1.76	1.48	16	28	32	21
Red clay	2.08	1.81	19.26	26	38.95	21.2

*ρ* = Natural density (g/cm^3^); *ρ*_d_ = Dry density (g/cm^3^); *W*_*n*_ = Natural water content (%); *W*_*s*_ = saturated moisture (%); *W*_L_ = Liquid limit (%); *W*_p_ = Plastic limit (%).

### Testing apparatus

Extensive literatures have documented that the residual shear strength ($\tau_{r}$) of slip zone soil is an important parameter for analysis and assessing the stability of slopes^27–29^. Methods currently used to measure the residual shear strength mainly include triaxial compression tests, reversal direct shear tests^30,31^ and ring shear tests^32–34^. By now, it has also been found that the use of the triaxial compression tests to determine residual shear strength of soil is not a first choice^35^. Compared with triaxial compression tests and direct shear test, ring shear tests enable simulating characteristics of slip zone soil in field with a large shearing displacement and keeping the shearing direction of specimen unchanged^36,37^.

**Figure 3:**
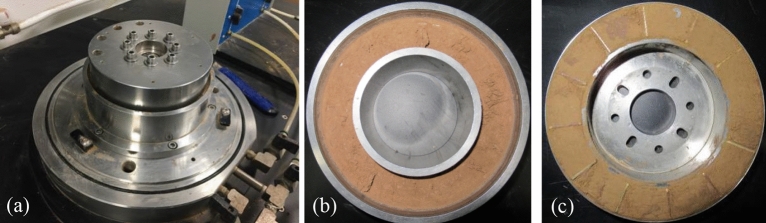
A sheared specimen. (**a**) Ring shear chamber; (**b**) the bottom platen with sheared soil; (**c**) the top platen with sheared soil.

Ring shear apparatus (SRS-150) manufactured by GCTS company of the United States was adopted in this research (Fig. 3a). In experimental process, the specimen is consolidated under a specific value of normal stress until the required consolidation was achieved. Then, the consolidated specimen is subjected to shearing by rotating the lower half of the shear box to obtain the shear strength and deformation of the specimen, while the upper half is fixed. The shear box has the inner diameter of 100 mm, the outer diameter of 150 mm (Fig. 3b), and the sample height of 25 mm. The ring shear rate can be controlled in the range of 0.001°/min to 360°/min. In this study, the shear plane on its upper half of the platen was used to analyze (Fig. 3c).

### Testing procedure

As mentioned above, the variation in the moisture content, normal stress and shear rate would result in the change in the mechanical properties of soils. To examine the effect of the variation of moisture content, normal stress and shear rate on the shear characteristics of soils, nine groups of three remolded specimens each have been prepared for the shear tests (Table 2). For each kind of soils (loess, red clay and LRCI), one group was tested at the normal stress of 200 kPa, shear rate of 1 mm/min (w = 12%, 20% and 28%), while the other two groups were tested at the moisture content of 12% and shear rate of 1 mm/min ($\sigma_{n} = 100,\,200\,{\text{and}}\,{300}\,{\text{kPa}}$), at the moisture content of 12% and normal stress of 200 kPa ($v = 0.01,\,0.1\,{\text{and}}\,1\,{\text{mm/min}}$), respectively. According to the physical parameters of soils listed in Table 1, the maximum and natural water content of Lishi loess are approximately 28.0% and 16% respectively, which means the water content of soils in the field varies widely and may exert considerable control on the mechanical properties of soils. Samples with moisture contents of 12% (less than 16%), 20% and 28% (equals to saturated water content) were prepared in this study.

**Table 2:** Ring shear test scheme.

$w$(%)	$\sigma_{n}$(kPa)	$v$(mm/min)
12	200	1
20		
28		
12	100	1
	200	
	300	
12	200	0.01
		0.1
		1

**Figure 4:**
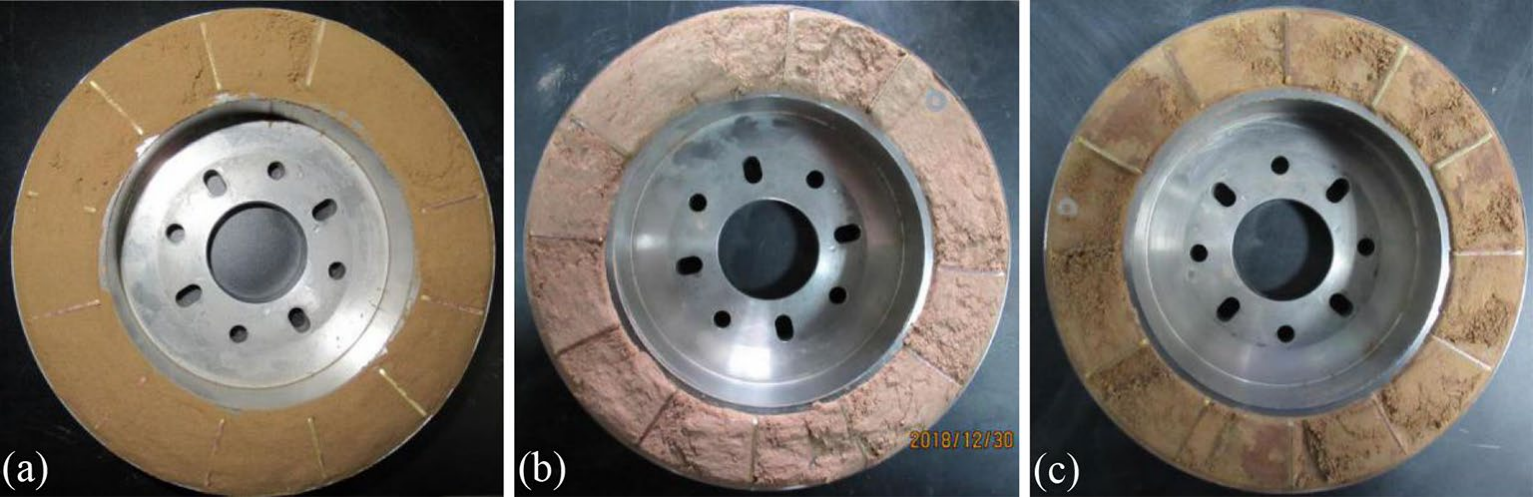
Shear planes of specimen after ring shear tests. (**a**) Loess; (**b**) red clay; (c) LRCI.

The sample for each test was prepared by the following procedures: the samples were packed in plastic bags and kept in a sealed container for about 48 h to allow water fully migrated within the specimen. To study the mechanical properties of LRCI sample, it is necessary to prepare an artificial shear plane of the loess-clay interlaminar. In this study, an artificial shear plane of loess-clay interlaminar was prepared by following procedures: Firstly, the red clay is placed in the shear box and consolidated using a specific normal stress according to the testing scheme (Table 2). The consolidated specimen was sheared until soil sample forms a continuous annular shear plane. Secondly, the upper shear box was removed and the red clay in the lower shear box was retained (see Fig. 3b). And then the red clay in the upper shear box was cleaned, the same thickness of loess was deposited into the upper shear box to ensure that the shear plane is located at the junction of red clay and loess while re-shearing. Finally, the upper shear box was installed again, and the loess-red clay specimen was consolidated under the original normal stress level. Thus, LRCI specimen with the fixed shear plane is prepared. During ring shear tests, loess samples and red clay samples are weighed separately and then placed into the shear chamber, and then the axial load is applied to the samples to achieve a required consolidation. Subsequently, the consolidated specimen is sheared until the residual state (i.e., the shear resistance value was kept at a constant value without further changing with progress of shearing) is achieved. Figures 4a–c show shear planes of three kinds of specimen after ring shear tests.

### Verification of test stability

**Figure 5:**
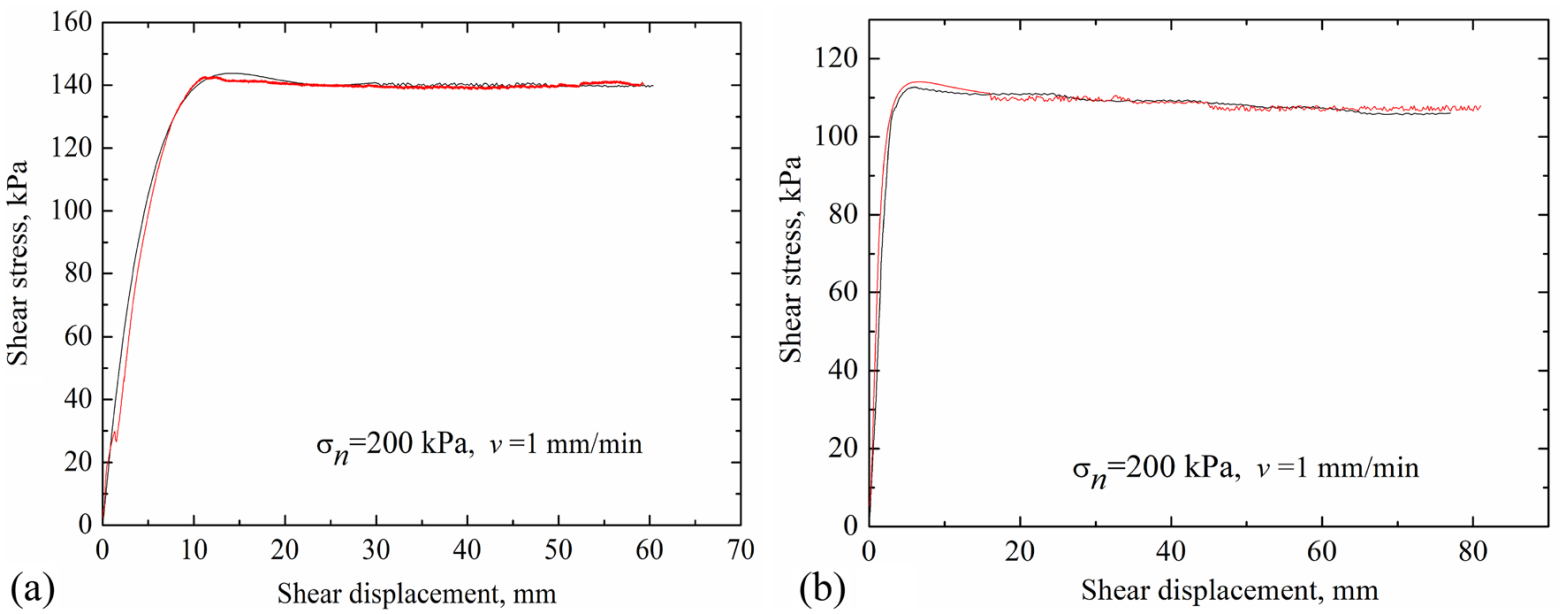
Verification of stability of the ring shear test. (**a**) Saturated loess; (**b**) saturated red clay.

To test the stability of the device, two groups of comparison tests were performed, using saturated loess soils and saturated red clay at the normal stress of 200 kPa, shear rate of 1 mm/min. The results are shown in Fig. 5. Figure 5a compares the results of remolded specimens of saturated loess soils, while Fig. 5b depicts the results of remolded specimens of saturated red clay. As can be seen in Fig. 5a,b, for the saturated loess soils and red clay, the stress-displacement curves of two sets of comparative tests are almost identical, indicating that the data obtained using the device is accurate and repeated tests with a good stability and high precision can be ensured by using this device.

## Results

**Figure 6:**
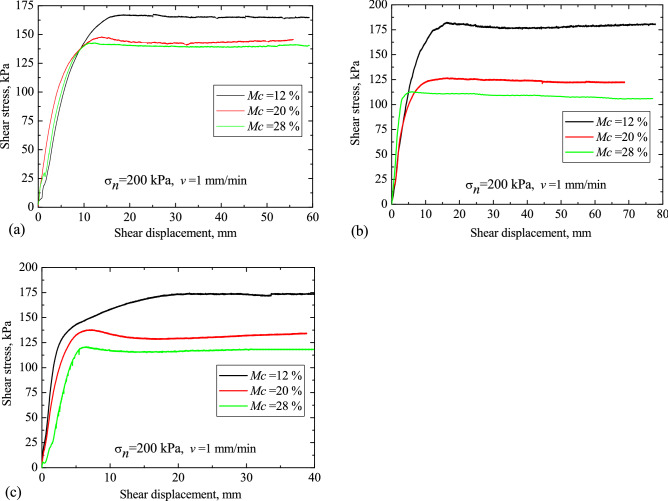
Stress–displacement curves under different moisture contents. (**a**) Loess; (**b**) red clay; (c) LRCI.

**Figure 7:**
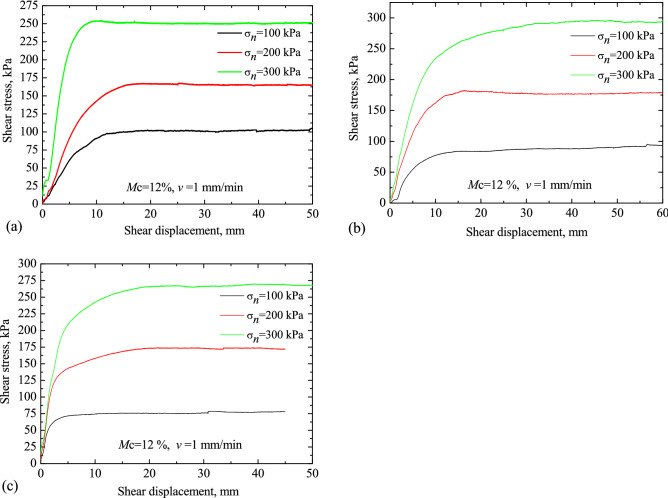
Stress–displacement curves under different normal stress. (**a**) Loess; (**b**) red clay; (**c**) LRCI.

**Figure 8:**
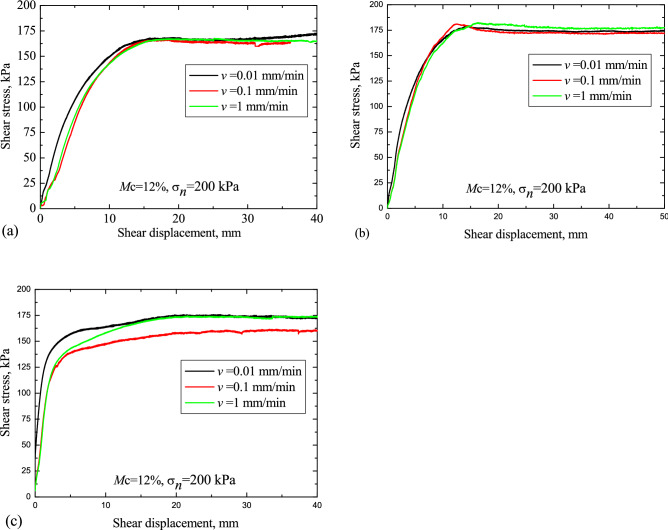
Shear stress versus shear displacement. (**a**) Loess; (**b**) red clay; (**c**) LRCI.

**Figure 9:**
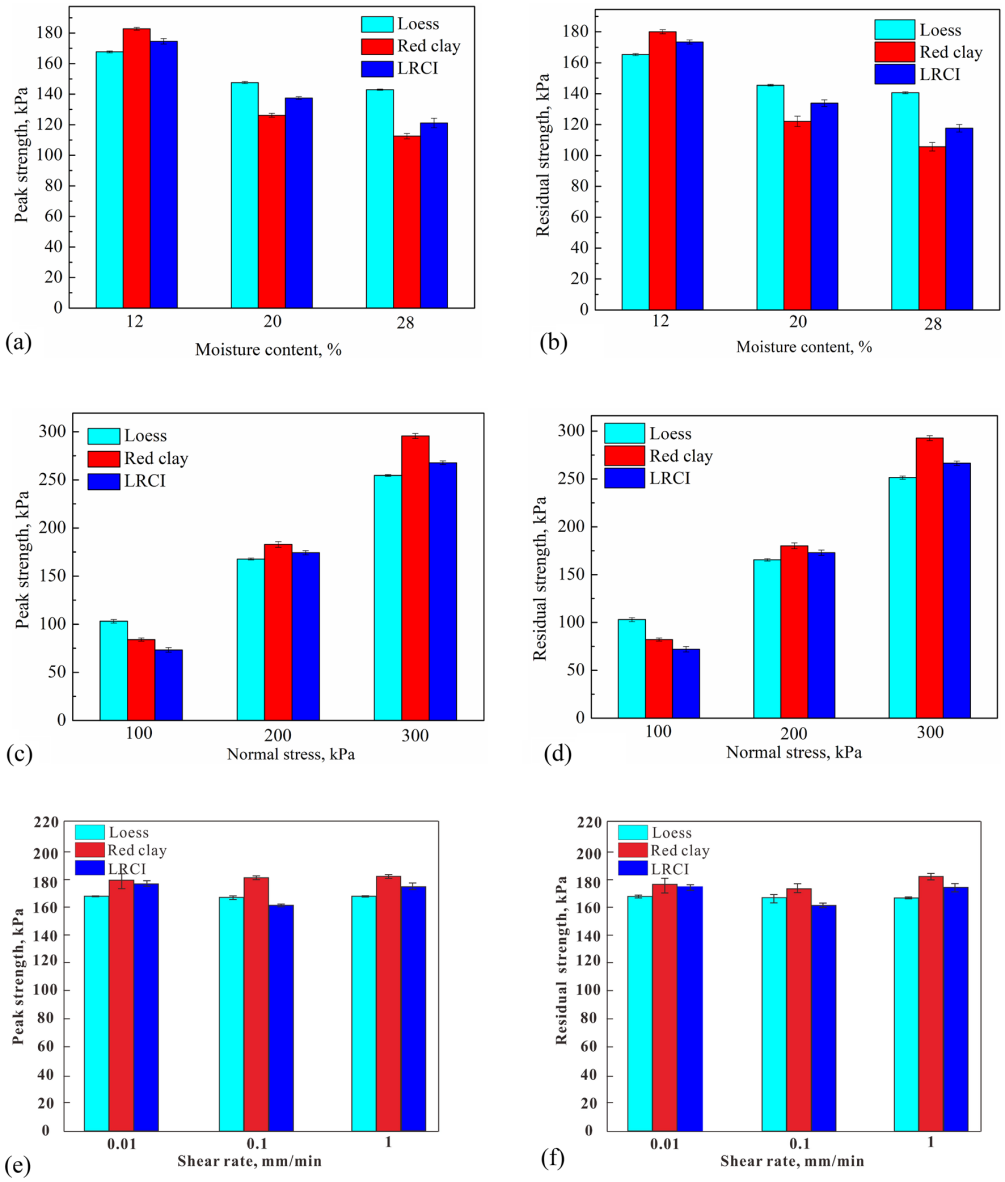
Histograms depicting peak strength and residual strength. (**a**,**b**) with moisture content; (**c**,**d**) with normal stress; (**e**,**f**) with shear rate. Error bars represent standard error (n = 3).

The typical shear stresses for loess, red clay and LRCI specimen are plotted in Figs. 6, 7 and 8, respectively, against the shear displacement. In addition, according to the stress-displacement curves, histograms depicting the peak strength (denoted as $\tau_{p}$) and residual strength (denoted as $\tau_{r}$) of soils are shown in Fig. 9. In current study, residual strength was determined following Salih et al.^38^ who suggested that the residual strength is attained if a relatively constant shear stress is measured for more than half hour. However, a constant value of residual strength was not observed due to the stress fluctuation in the shearing process, even the specimen was sheared at a large displacements. Thus, over a certain period (more than half hour), the lowest shear resistance that was kept at a roughly stable value was adopted as the residual strength in this study.

### Shear behavior

Moisture content has been recognized as a fundamental factor affecting shear properties of soil^21,39,40^. The experimental results of three samples with moisture contents of 12%, 20% and 28%, are selected to illustrate the effect of the moisture content on the shear behavior of soil samples. When $\sigma_{n}$ = 200 kPa and $v$ = 1 mm/min, the typical shear behavior of specimens is shown in Fig. 6, where, the shear stress is plotted against the shear displacement. As shown in Fig. 6, the response curves are almost similar for all samples for testing. However, in comparison to the loess and red clay (Fig. 6a,b), the shear resistance of LRCI reaches its peak after a larger displacement (more than 10%), and then shows a small decrease with further shearing (Fig. 6c). Meanwhile, it can be noted that for a specific specimen, the peak shear strength as well as the residual shear strength decreases with increasing moisture content (Fig. 9a,b)). Moreover, among three kinds of specimen, the moisture content has the greatest influence on the $\tau_{p}$ and $\tau_{r}$ of red clay, followed by LRCI specimen and the loess specimen is least affected by moisture content. To be more specific, an increase in Mc (from 12 to 28%) of red clay causes a considerable reduction in $\tau_{r}$(from 180.02 kPa to 105.76 kPa). Comparatively, for LRCI specimen, $\tau_{r}$ tends to decrease from 173.42 kPa to 117.68 kPa when Mc increases from 12 to 28%, decreasing about 32%. And for loess specimen, $\tau_{r}$ decreased from 165.28 kPa to 140.66 kPa with Mc increased from 12 to 28%, decreasing approximately 15% (Fig. 9b).

However, it is seen that, for a given moisture content, the comparison in $\tau_{r}$ among three kinds of specimen is complicated (Fig. 9b). For example, at Mc of 12%, $\tau_{r}$ of red clay (180.02 kPa) is greater than that of LRCI (173.42 kPa), and $\tau_{r}$ of loess (165.28 kPa) is the minimum. Nevertheless, $\tau_{r}$ of loess (145.49 kPa) is slightly higher than that of LRCI (133.89 kPa) with Mc of 20%, and the minimum $\tau_{r}$ of red clay is obtained at Mc of 20%, reaching about 122.12 kPa. In addition, the $\tau_{r}$ of loess, LRCI and red clay is 140.66 kPa, 117.68 kPa and 105.76 kPa, respectively, when Mc is 28%. The experimental results herein suggested that when moisture content is above a certain value, the shear strength of the red clay and LRCI will be greatly less than that of loess soils.

**Figure 10:**
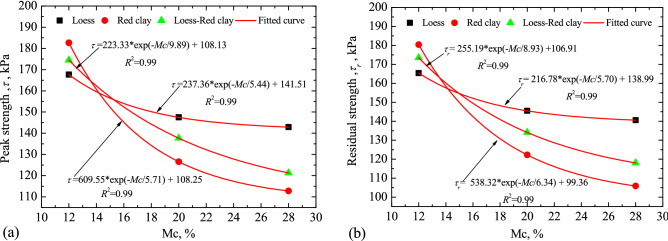
Relationship between shear strength and moisture content. (**a**) Peak shear strength; (**b**) residual shear strength.

Based on the experimental data (Fig. 9a, b), the relationships between shear strength and moisture content are shown in Fig. 10.

By fitting the experimental data in Fig. 9a, the relationships between the peak shear strength and the moisture content for loess, red clay and LRCI were expressed as follows, respectively:

1 $$ \tau_{p} = 237.36e^{{( - {\text{Mc}}/5.44)}} + 141.51,\quad 12 \le {\text{Mc}} \le 28 $$

2 $$ \tau_{p} = 609.55e^{{( - {\text{Mc}}/5.71)}} + 108.25,\quad 12 \le {\text{Mc}} \le 28 $$

3 $$ \tau_{p} = 223.33e^{{( - {\text{Mc}}/9.89)}} + 108.13,\quad 12 \le {\text{Mc}} \le 28 $$

In addition, the variation in the residual shear strength with moisture content of loess, red clay and LRCI were written as follows by fitting the experimental data in Fig. 9b:

4 $$ \tau_{r} = 216.78e^{{( - {\text{Mc}}/5.70)}} + 138.99,\quad 12 \le {\text{Mc}} \le 28 $$

5 $$ \tau_{r} = 255.19e^{{( - {\text{Mc}}/8.93)}} + 106.91,\quad 12 \le {\text{Mc}} \le 28 $$

6 $$ \tau_{r} = 538.32e^{{( - {\text{Mc}}/6.34)}} + 99.36,\quad 12 \le {\text{Mc}} \le 28 $$

It can be seen from Eqs. (1–6) that: both the peak strength and the residual strength decreased with the increase of moisture content for all specimens, consistent with the observations from tests reported in other studies^13,31^. Meanwhile, it has been shown that the shear resistance of red clay samples is more sensitive to the variation of moisture content compared with that of loess and LRCI samples. The correlations between the shear strength and moisture content were obtained and shown in Eqs. (1)–(6), which can be used to quantitatively understand the triggering mechanism of such kind of landslides and to predict the landslides evolution in future by combining with numerical simulation methods.

The relationship between shear stress and shear displacement under different normal stresses is shown in Fig. 7. It can be found that: (1) as normal stress increases, both the peak shear strength and the residual shear strength shows an increasing tendency; (2) LRCI specimen is greatest affected by the normal stress, followed by red clay, and the loess specimen is least affected by the normal stress. To be more specific, the magnitude of $\tau_{r}$ of LRCI specimen at normal stress of 300 kPa (266.47 kPa) is 3.7 times the magnitude of $\tau_{r}$ at normal stress of 100 kPa (72.03 kPa). As for red clay specimen, the $\tau_{r}$ at normal stress of 300 kPa (292.76 kPa) equals to 3.56 times the $\tau_{r}$ at normal stress of 100 kPa (82.15 kPa). Comparatively, an increase from 100 to 300 kPa of normal stress of loess specimen result in an increase of the $\tau_{r}$ from around 103.01 kPa to 251.39 kPa, i.e., more than 2.4 times (Fig. 9d). This study shows that residual strength increases with normal stress, a finding that agrees with a number of previous studies^41,42^.

By now, it has been found that the residual shear strength of soil may be positively dependent, negatively dependent on shear rates or unrelated with shear rates^32,33,43–45^. The experimental results shown in Fig. 9e,f revealed that the effect of shear rate on the shear strength is almost negligible, which is consistent with the previous studies^33,45,46^, in which they reported that shear rate has little effect on the shear strength with shear rate lower than 10 mm/min.

### Macroscopic observation in the morphology of LRCI shear surface

**Figure 11:**
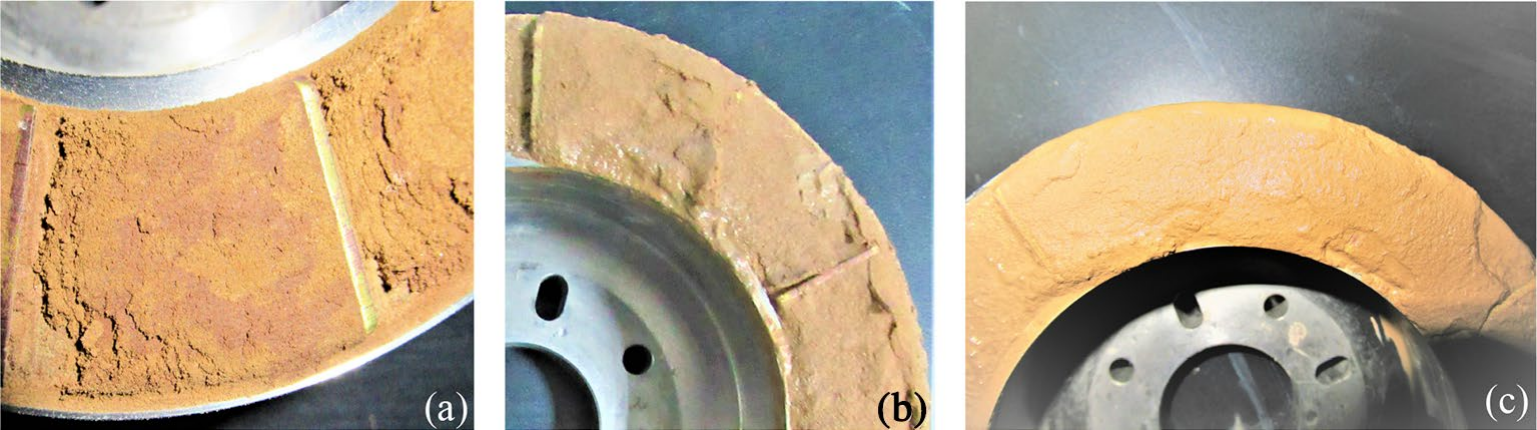
Macroscopic morphology of LRCI Shear Surface with different moisture contents **(**$\sigma_{n}$ = 200 kPa, $v$ = 1 mm/min). (**a**) Mc = 12%; (**b**) Mc = 20%; (**c**) Mc = 28%.

**Figure 12:**
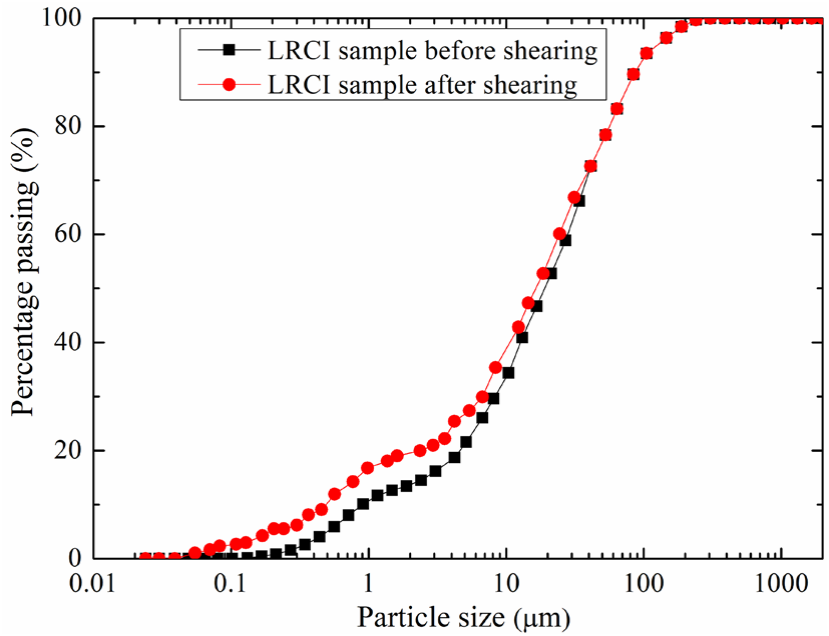
Particle size distribution of LRCI shear surface before and after shearing.

The macroscopic morphology of LRCI shear surface at different moisture contents is shown in Fig. 11. A localized water accumulation was developed within the shear plane at the moisture content of 20% (Fig. 11b). Meanwhile, a high degree of liquefaction phenomenon was clearly observed at the moisture content of about 28% (close to the saturation degree) in Fig. 11c. Other studies^10,33,47^ similarly reported the occurrences of the liquefaction in shear plane of saturated loess samples. Such phenomenon can be explained as follows: during the shearing process, a smooth shear plane develops within the LRCI specimen (Fig. 11a), which is consistent with the conclusion reported by some researchers^37,48^ that a smooth shear plane appears in the clay-rich soil when the shear displacement is large enough^29,35^. As moisture content increases, the soil matrix suction decreases gradually^49^, and eventually more free water accumulates on the shear plane, causing liquefaction of the soil sample, which also lubricates the soil particles near shear plane and thus reduces the frictional bite force between the soil particles. Therefore, the shear strength of the sample gradually decreases as water content increases (Fig. 9a,b). It has been acknowledged that the occurrence of a loess landslide is usually attributed to static liquefaction^50,51^. The experimental results indicated that the LRCI soils with a relatively high degree of saturation shows the potential of localized liquefaction which may be related to the occurrence of a loess-red clay landslide with high-speed and long- run out. The particle size distribution of LRCI shear surface, determined using a Mastersizer 2000 (UK) laser particle size analyzer, shows that compared with specimen prior to shearing, the fine particle content increased after shearing (Fig. 12).

### Change in the microstructure of the shear surface with moisture content

**Figure 13:**
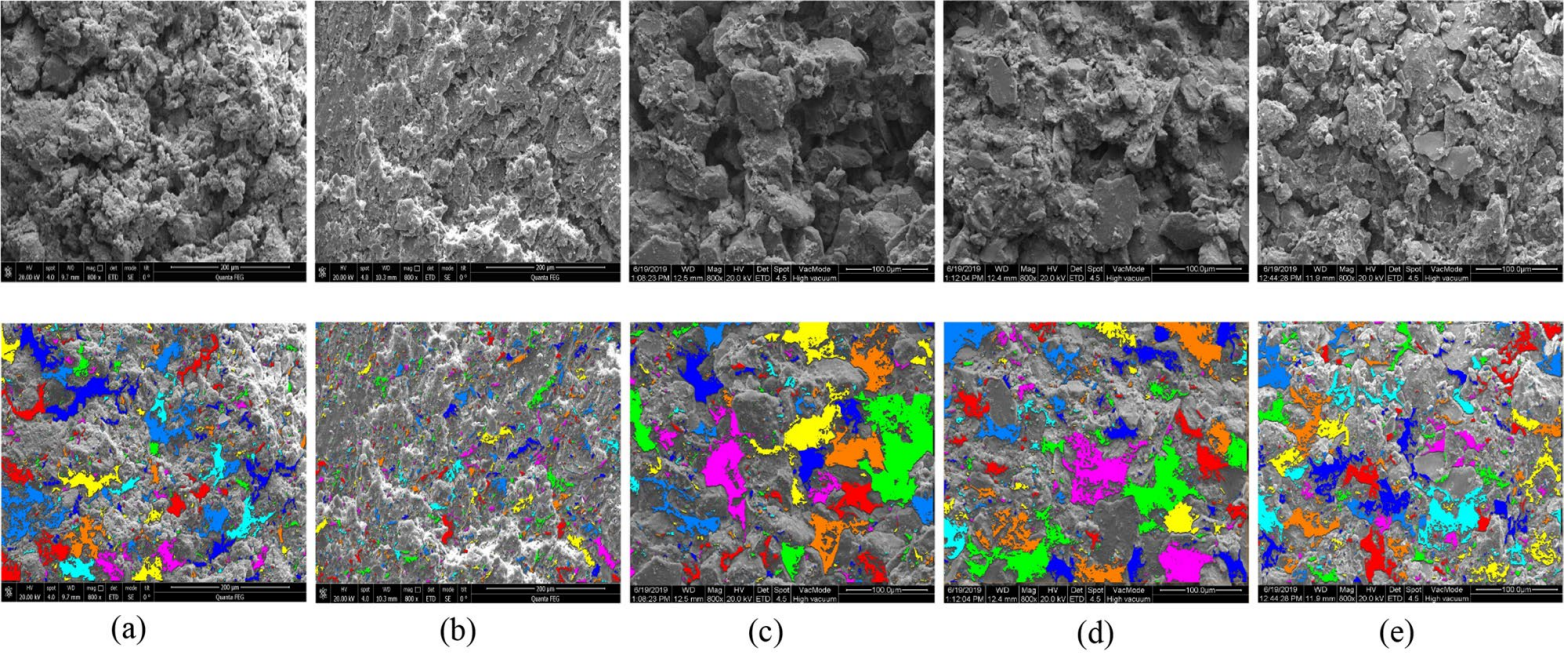
SEM images of shear surface with different moisture contents (800 × , $\sigma_{n}$ = 200 kPa, $v$ = 1 mm/min). (**a**) Loess, Mc = 12%; (**b**) red clay, Mc = 12%; (**c**) LRCI, Mc = 12%; (**d**) LRCI, Mc = 20%; (**e**) LRCI, Mc = 28%.

It should be noted that the significance of microstructure in evaluation the deformation and shear strength characteristics of soil^38,52^. Furthermore, the change in the microstructure of slip zone soils during the shearing process is complicated^53^ and the macroscopic mechanical properties of soils could be directly related to its microstructure^54,55^. Therefore, to exemplify the variation in the microstructure of samples, scanning electron microscopy (SEM) tests were conducted on shear planes of specimens at different moisture contents levels. After the ring shear tests, air-drying shear planes with area of about 1 mm^2^ were used to conduct SEM tests. The SEM images of loess, red clay and LRCI samples are shown in Fig. 13a–e.

From Fig. 13a–e, it can be seen that the microstructure characteristics of shear surfaces obtained from three kinds of specimens with the moisture content of 12% are as follows: the structure of loess is mainly compose of macro-pores with various sizes (Fig. 13a). For the red clay dominated by minerals components such as montmorillonite and illite^7,10^, the flake microstructure appears within shear surface (Fig. 13b), which may be attributed to the rearrangement of flaky particles with obvious reorientation^13^. Nevertheless, LRCI is a mixture of loess and red clay granules, within which the contact between the flaky particles is not very tight due to the existence of pores (Fig. 13c) and flaky particles show some degree of preferred orientation. From a microscopic viewpoint, the LRCI sample (Fig. 13c) has more porosity than the red clay sample (Fig. 13b). Consequently, the attraction between the soil particles of LRCI sample becomes smaller, resulting in a reduction in shear strength (Figs. 9, 10).

With an increase in moisture content of LRCI specimen, the shear surface become smoother and the larger percentage of small-sized pores are observed (Fig. 13c–e). In addition, from the perspective of mineral composition, the flaky clay mineral particles in the LRCI sample are characterized by a strong adsorption capacity. Thus, the hydrated film is thickened and expanded^56^ when more water infiltrates into the LRCI sample. Consequently, the sliding friction is vulnerable to occurring between the granules, leading to a great reduction in the shear strength with increasing moisture content.

To quantitatively clarify the effect of moisture content on the micro-porosity parameters including surface porosity ($P_{{}}$) and Mean Pore Area ($\overline{S}_{i}$), Avizo9.0 software was adopted to process Fig. 13a–e following previous studies (Wong et al., 2018; Hu et al., 2018), with which the skeleton algorithm was applied to segment a single pore of the image (Suuronen et al., 2013; Sarkar and Siddiqua 2016) and the results are shown in Figs. 13a'–e'. Surface porosity, denoted by $P_{{}}$, is expressed as:

7 $$ P_{{}} = \frac{\Sigma N}{{N_{length} \times N_{width} }} $$

where $N_{length}$ and $N_{width}$ are the number of pixels of the long and short sides of the image, respectively, and $N$ is the number of pixels of the pore.

Mean Pore Area which is denoted as $\overline{S}_{i}$ , is given as:

8 $$ \overline{S}_{i} = \frac{{\sum {S_{i} } }}{{N_{p} }},\quad S_{i} = N_{i} \times L_{{{\text{vox}}el}}^{2} , $$

where $S_{i}$ is the area of the $i$th pore , $N_{p}$ is the total number of pores, $N_{i}$ is the number of pixels of the $i$th pore; $L_{voxel}$ is image resolution.

**Figure 14:**
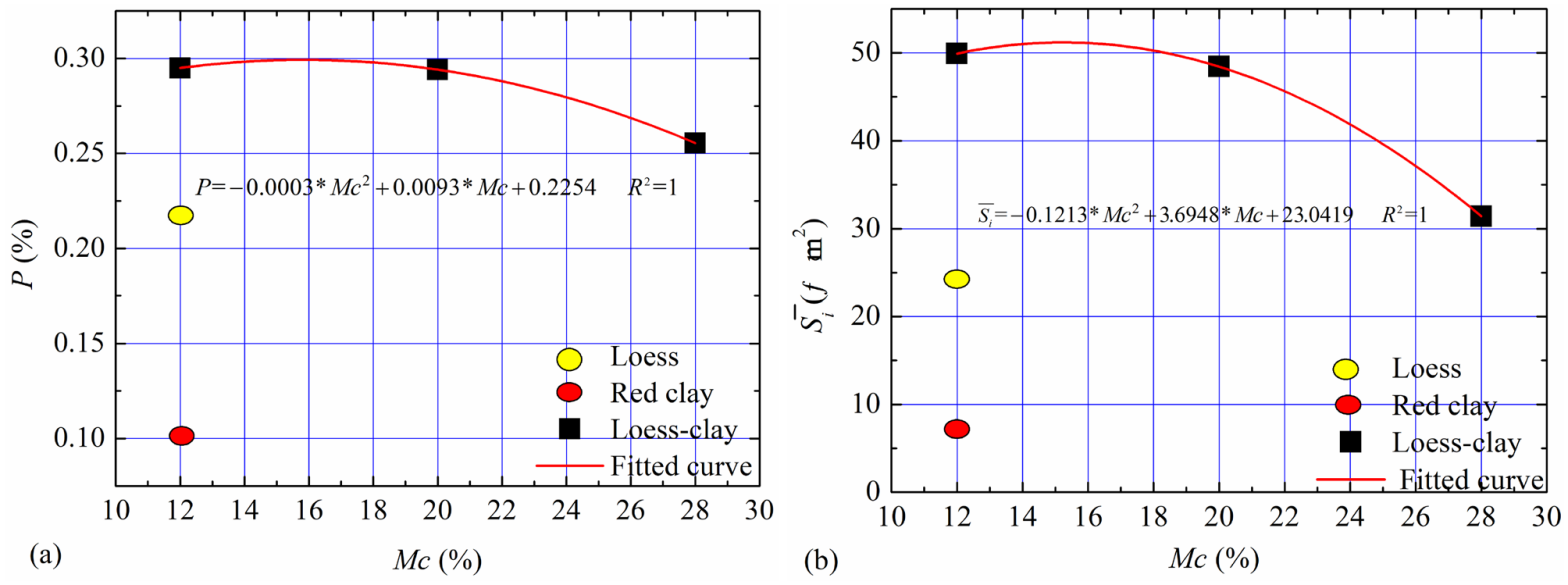
The micro-porosity parameters. (**a**) Surface porosity, (**b**) mean pore area.

To quantitatively clarify the effect of moisture content on the micro-porosity parameters of the LRCI specimen, the data in Fig. 14 were fitted as follows:

9 $$ P{ = } - 0.000{3}*Mc^{2} + 0.00{93}*Mc + 0.{2254}\quad 12 < Mc < 28 $$

10 $$ \overline{{S_{i} }} { = } - 0.{1213}*Mc^{2} + {3}.{6948}*Mc + {23}.0{419 }\quad 12 < Mc < 28 $$

The estimated surface porosity and mean pore area is listed in Table 3 and plotted in Fig. 14. As shown in Fig. 14, at the same moisture content (Mc = 12%), Surface porosity($P_{{}}$) and Mean Pore Area($\overline{S}_{i}$) of the LRCI sample is greatest while those values of the red clay sample are the minimum (Fig. 14). Additionally, moisture content plays a very significant role in the micro-parameters of LRCI specimen. For example, the surface porosity decreases from 48.919 to 31.42 $\mu {\text{m}}^{2}$ with moisture content ranging from 12 to 28%, and a drop in the mean pore area of nearly 13% (from 0.295% to 0.255%) for LRCI specimen with moisture content ranging from 12 to 28% is observed.

**Table 3:** The micro-porosity parameters.

Sample type	Surface porosity (%)	Mean pore area (μm^2^)
Loess (Mc = 12%)	0.217	23.966
Red clay (Mc = 12%)	0.099	7.115
LRCI (Mc = 12%)	0.295	48.919
LRCI (Mc = 20%)	0.294	48.434
LRCI (Mc = 28%)	0.255	31.428

## Discussion

### Triggering mechanism of loess-red clay landslides

The occurrence of the loess-red clay landslides may be associated with the steep slope, slope structure, physical and mechanical properties of the loess, red clay and the nature of the loess-red clay contact. Based on the geological setting of the slope and the variations of shear resistance of the loess, red clay and LRCI samples, the triggering mechanism of a landslide occurred along a contact between loess soils and red clay can be explained as follows: the steep topography of the slope with a great gradient (Fig. 1), indicates that the slope is susceptible to instability. In the season with intense precipitation, precipitation easily infiltrates into the loess along the upper cracks when the overlying loess is characterized by a great height difference and free surfaces (Fig. 1). Compared with loess soils, the red clay is poorly permeable^10^, which easily causes the water accumulation on the contact between loess and red clay (Fig. 11). Consequently, the contact surface between loess and red clay layer is softened by water infiltration^6,18^, which causes the mechanical behavior of the contact surface to significantly decrease (Fig. 9) and to develop a softened interlayer (which also be called as weak zone). From a micro perspective, with increase in moisture content due to infiltration into the weak zone, cementing materials such as organic matter and soluble salts in LRCI soils gradually decrease and even disappear, and some clay aggregations are softened or disintegrated^57^. In addition, further increase in the moisture content caused by heavy rainfall would result in the dissolution and leaching of mineral materials such as CaCO3 (also serving as cementing material) as well^58^. With the disintegration of the cementing materials, the space for soil particle shearing increases. Eventually, the skeleton soil particles within shear zone are rearranged and reoriented and thus a relatively closely particle structure of LRCI is reached (Fig. 13). Furthermore, the disintegrated cementing materials also rearrange due to the adjustment of soil particles. Consequently, some larger pores in LRCI soils are transformed into smaller pores (Figs. 13, 14). Therefore, the initial open structure of LRCI soils becomes a relatively uniform and close structure^59^in which the permeability of soils within shear zone decreased dramatically. Accordingly, the pore water moves into the small-sized pores and the built-up excess pore water pressure is difficult to be dissipated due to a close structure of small-sized pores. Owing to the low permeability, high pore-water pressure built up within the shear zone and the increase in the fine particle content (Fig. 12), the LRCI soils with a high saturation degree shows the potential for the localized liquefaction within shear zone (Fig. 11). Once the shear resistance reached up to the shear strength of weak zone surface, the shear surface develops into the sliding surface and the overlying strata in the slopes slides along the sliding surface and eventually leads to the slope failure.

### The limitation of experiment and the future work

Firstly, under the effect of gravity of the overlying loess soils, the shear surface of the slip zone soil of the loess-red clay landslide is not actually the circular plane shown in the current study, but a more complicated curved surface. Thus, ring shear tests with more practical curved shear surfaces would be explored with an advanced equipment and technology in future. Secondly, due to the difficulties in the sample preparation, only three sets of ring shear tests with different moisture contents were conducted. Thus, the quantification of correlation given can only describe the relationship between the shear strength and moisture contents in a small range. Therefore, a large number of ring shear tests with moisture contents in a wide range are needed to be done in the future. In addition, the composition of slip zone soil is not the simple materials contacting between loess and red cl ay in some practical engineering, but a mixture soil of loess and red clay with a certain thickness. Furthermore, the mixing ratio of such mixed soil tends to vary at different locations in the slip zone^23^ with influencing factors such as clay content^40^, clay mineral composition^60^, shape and gradation of soil particles^13^, microstructure^61^ and stress^16^. Therefore, the determination of shear strength of slip zone soils is complicated and challenging^29,62^. Further experimental studies on slip zone soil of loess-red clay landslides, which focuses on considering limitations of the experiments mentioned above, would be performed in our future research to examine the validity of the above-mentioned test results in the movement prediction of loess-red clay landslides.

## Conclusions

Basing on the results of ring shear tests on the loess soils, red clay soils taken from a typical loess-red clay landslide in Loess Plateau Hinterland and LRCI specimens, the mechanical properties of slip zones between loess and red clays as well as the triggering mechanism of loess-red clay landslides were discussed. The following conclusions were drawn:

(i) Both the peak and residual strength of loess, red clay and LRCI specimen decrease exponentially with increasing moisture content. Among which, the moisture content has the greatest influence on the peak and residual strength of red clay, followed by the LRCI specimen, and the loess specimen is least affected. In addition, with the moisture content above a certain value, shear resistance of LRCI and red clay is dramatically less than that of the loess specimen.
(ii) Ring shear tests on loess soils, red clay and LRCI specimens showed that the peak and residual strength of soils is dependent upon normal stress. There is a positive correlation between the normal stress and increase in shear resistance. In addition, LRCI specimen is greatest affected by normal stress, followed by red clay, and the loess specimen is least influenced by the normal stress.
(iii) The macroscopic morphological characteristics of shear surface obtained from the LRCI specimens demonstrated that a localized water accumulation was built up within the shear surface as the water content increases to some extent, and a high degree of liquefaction developed within specimen when the moisture content reaches to the saturated degree., which shows the potential of localized liquefaction which may be related to the occurrence of a loess-red clay landslide.
(iv) The microstructural testing results show that with an increase in moisture content of LRCI specimen, the shear surface become smoother and the larger percentage of small-sized pores are observed. Accordingly, the pore water moves into the small-sized pores and the built-up excess pore water pressure is difficult to be dissipated due to a close structure of small-sized pores. Owing to the low permeability, high pore-water pressure built up within the shear zone and the increase in the fine particle content, the LRCI soils with a high saturation degree shows the potential for the localized liquefaction within shear zone, which further provides a scientific explanation for the occurrence of loess-red clay landslides with high-speed and long run out.
